# Personality Traits Associated with the Risk of Exercise Dependence in Ultraendurance Athletes: A Cross-Sectional Study

**DOI:** 10.3390/ijerph20021042

**Published:** 2023-01-06

**Authors:** Marion Remilly, Benoit Mauvieux, Joffrey Drigny

**Affiliations:** 1 Normandie Univ, UNICAEN, CHU de Caen Normandie, Service de Médecine du Sport, 14000 Caen, France; 2 Normandie Univ, UNICAEN, INSERM, COMETE, GIP CYCERON, 14000 Caen, France; 3 Normandie Univ, UNICAEN, CHU de Caen Normandie, Normandie University, Service de Médecine du Sport, Service de Médecine Physique et de Réadaptation, INSERM, COMETE, GIP CYCERON, 14000 Caen, France

**Keywords:** ultraendurance sport, exercise addiction, personality, neuroticism

## Abstract

Exercise dependence (ED) is common in endurance athletes and can lead to physical and psychological distress with various health effects. We designed a prospective cross-sectional study to investigate the personality traits associated with ED among ultraendurance athletes. A total of 507 participants (41.6 (9.8) years, men: 73.7%) completed (1) a screening questionnaire about sociodemographic data, sporting habits, and healthcare data, (2) the Exercise Dependence Scale-Revised (EDS-R, 21 items scored from 1 (never) to 6 (always), 7 subscales), (3) the Big Five Inventory (BFI), and (4) 2 items of the SCOFF (Sick-Control-One Stone-Fat-Food) questionnaire regarding possible eating disorders. Based on the EDS-R scores, 37 (7.3%) participants were at risk for ED (scores ≥ 5/6 on ≥3 subscales), 366 (72.2%) were nondependent but symptomatic (scores ≥ 3/6 on ≥3 subscales), and 104 (20.5%) were asymptomatic. Participants with ED had a greater training volume and a higher prevalence of possible eating disorders. A higher level of neuroticism was associated with increased EDS-R scores (r = 0.294; *p* < 0.001), with significantly higher scores in the ED group (F = 14.50, *p* < 0.001). The association between neuroticism and ED was not moderated by the presence of eating disorders. These findings will help to screen ultraendurance athletes at risk for ED and optimize their care.

## 1. Introduction

The practice of regular physical activity, including exercise, has numerous benefits for mental and physical health [1,2]. Thus, public health authorities and experts encourage increased participation in physical activity and exercise for the prevention of chronic diseases associated with physical inactivity and sedentary behavior [3]. Additionally, regular physical activity is key for the prevention and/or treatment of mental health problems [4,5,6], and exercise-based interventions have been proposed as a treatment for addiction and substance use disorders [7,8,9].

However, excessive exercise needs to be distinguished from exercise that occurs at a high frequency and should be distinct from a healthy habit in that excessive exercise occurs to the detriment of physical health, quality of life, and social responsibility [10]. Indeed, exercise dependence (ED) is characterized by behavioral, psychological and physiological symptoms similar to those of substance use disorders or behavioral addictions [11,12] and by engagement in any form of exercise despite negative consequences [13]. ED has been described as a “positive” addiction due to the large benefits of physical activity and regular exercise [14]; however, there is substantial evidence that ED should be considered a behavioral addiction with detrimental effects and potential coexisting disorders [15].

There is no clear consensus on the definition of ED, and the most accepted characterization is based on the criteria for common substance use disorders [16,17]. According to the Diagnostic and Statistical Manual of Mental Disorders (5th edition, DSM-5), substance use disorder is characterized by the following symptoms: tolerance (increased amount of exercise to get the desired effect), withdrawal (negative effects in the absence of exercise), loss of control (unsuccessful efforts to control exercise), intention effects (exercising longer or at a higher intensity than intended), duration (a great deal of time dedicated to exercise), a reduction in other activities, hazardous behaviors (harmful exercise for mental and physical health), failure to fulfill obligations, social/interpersonal problems, and continuance (continuing to exercise despite detrimental effects) [18]. However, unlike gambling, ED has not yet been designated a “nonsubstance-related disorder” in the “Substance-related and Addictive Disorders” category of the DSM-5, mostly due to insufficient peer-reviewed evidence [19]. Additionally, substance dependence is characterized in the International Classification of Diseases (11th edition, ICD-11) by the presence of two or more of three composite guidelines among (1) impaired control over substance use, (2) substance use becoming an increasing priority in life, and (3) physiological features as manifested by tolerance, withdrawal symptoms, or repeated use [20]. Additionally, the ICD-11 included non-substance addiction disorders in the grouping of impulse control disorders, which should be defined by the repeated failure to resist an impulse, drive, or urge to perform an act that is rewarding to the person (at least in the short term), despite longer-term harm [21]. However, ED is not recognized in the ICD-11.

There is a large body of literature supporting that ED can be either a primary addiction or a secondary coexisting disorder associated with other mental or medical conditions [22,23], especially eating disorders [24,25]. Recent knowledge suggests that the etiology of ED differs depending on the presence or absence of an eating disorder [26], but there is no valid or common conceptualization of compulsory exercise in individuals with eating disorders [27]. Additionally, recent evidence has shown that the risk of ED is associated with coexisting mental disorders such as anxiety and depression or obsessive-compulsive disorders [28] as in other behavioral addictions, but there is uncertainty about the causal factors that may underlie ED [29].

If ED is not restricted to the consideration of high-volume training, it may contribute to extreme volumes and/or intensities of long-term exercise training and be associated with health issues. The consequences of overtraining are widespread, negatively affecting several physiological systems, including neuroendocrine, immunological, neuromuscular and skeletal, and cardiovascular disorders [30,31]. Overtraining could also result in several negative psychological disturbances, such as increased depression, low motivation, anger, and eating disorders [30,31]. Interestingly, Golshani et al. recently conducted a comparative study exploring the prevalence of mental health issues among heavy (18–22 h/week) and light exercisers (1–6 h/week) [32]. They found that heavy exercising might be associated with more mental health issues, and that lower mental toughness and more sleep disturbances could predict worse mental health issues in heavy exercisers.

The prevalence and characteristics of ED have been largely studied in endurance sports due to the high volume and intensity of training, with high susceptibility to developing ED [33,34,35]. Among endurance sports, ultraendurance sports are defined as events lasting at least 6 h [36], and the sports concerned are mostly trail running, ultramarathons, ultracycling, Ironman triathlons, open-water swimming, and cross-country skiing. The practice of ultraendurance sports has become increasingly popular in recent years, mostly for recreational athletes [37,38,39]. Additionally, there is extensive evidence that ultraendurance races and regular ultraendurance training may lead to long-term health problems, including cardiovascular, respiratory, musculoskeletal, renal, immunological, gastrointestinal, neurological, and skin problems [40,41]. As ED is characterized by continuous exercise despite significant and interfering physical problems that are likely to have been caused by intense exercising, it is essential to better understand the continuum and psychological and/or interactional mechanisms underlying ED in ultraendurance athletes to prevent both mental health issues and related health problems [42].

ED is a complex and multifaceted behavior encompassing several psychological and physiological characteristics and resulting from the interaction of a multitude of personal and situational factors [43]. To better understand the behavioral tendencies and personality features underlying addiction, numerous studies have explored their association with personality traits. Personality is defined by an individual’s set of stable traits and behaviors, which are the expression of their ways of living, relating to others and perceiving their own person in association with their environment [44]. Personality aspects are grouped into personality traits, which are relatively enduring patterns of thoughts, feelings, and behaviors that reflect a person’s tendency to react in certain ways under certain circumstances and that demonstrate both continuity and change over time [45]. These studies have highlighted specific and similar personality traits among individuals with substance use disorders or behavioral addictions [46,47]. Most studies have used the Big Five model to describe the broad traits that serve as building blocks of personality in individuals with substance and behavioral addictions [48,49,50]. These five primary personality traits are extraversion, agreeableness, openness, conscientiousness, and neuroticism [51]. Individuals with substance use disorders or dependence have mostly high neuroticism and low openness and/or agreeableness [49]. Some studies have explored the association between personality traits and ED [52,53,54]. In 2017, Bircher et al. conducted a systematic review with conflicting results and concluded that if personality factors were involved in exercise addiction, the current knowledge is insufficient to draw a specific profile of the personality of a person with exercise addiction, and further studies with more representative samples are needed [55].

Although personality traits have been identified in ultraendurance athletes (high extraversion and openness to experience) [56], little is known about the traits associated with ED in this population. Given the prevalence of ED in ultraendurance athletes with potential detrimental impacts on their mental and physical health, the identification of personality traits associated with the risk of ED could help physicians and healthcare workers better identify athletes before they experience symptoms of dependence.

The present study aimed to investigate the personality traits associated with ED among ultraendurance athletes and identify the symptoms associated with the significant personality traits. The secondary objectives were to assess the prevalence of ED, test participant characteristics, and screen for eating disorders associated with ED in ultraendurance athletes.

Regarding our main objective, the hypotheses were that ED could be associated with personality traits similar to those of other behavioral addictions or SUD, with high neuroticism and low openness and/or agreeableness. Additionally, we hypothesized that the coexistence of possible eating disorders would interfere with the strength and level of association between personality traits and ED.

## 2. Materials and Methods

### 2.1. Design

This prospective cross-sectional study was conducted in an academic medical center from 1 April 2022 to 30 May 2022. The local research committee granted approval (Caen University’s Health Research and Ethics Committees COTECH: Ref. 3363), and the study protocol was registered in an international database (ClinicalTrials.gov NCT05348798). The study was conducted in accordance with the Declaration of Helsinki, and informed consent was obtained from all participants.

### 2.2. Population

The study population was all volunteer adult athletes (aged 18 years or older) who spoke French, had participated in an ultraendurance event, and had prepared for at least one ultraendurance event in the current year (e.g., events lasting at least 6 h). The disciplines considered were trail running, Ironman triathlons, ultracycling, ultradistance running or ultramarathons, ultradistance open-water swimming, swim-run races, multisport raids, and cross-country skiing. Participants were recruited via federal associations, ultraendurance event organizers, and social networks. The list of federations/event/social media groups targeted for the survey is presented in a Supplementary File (Table S1).

### 2.3. Measures

All participants completed a secure and anonymous web-based questionnaire that took approximately 20 min to complete. The questionnaires included a screening questionnaire about demographic data, healthcare, and sporting characteristics, the Exercise Dependence Scale-Revised (EDS-R), the Big Five Inventory (BFI), and two items of the SCOFF questionnaire to screen for eating disorders. A website version of the questionnaire was developed using the Google Forms platform. An introductory page provided all information regarding the study, participants’ rights, and ways to contact the researcher. All participants were informed that participation was voluntary and anonymous. A clear statement indicated that consent was assumed after checking a mandatory “I agree” button that provided access to the survey. The survey platform forced the completion of all questions before the respondent could move on, and all of the collected questionnaires were complete. Personal data (including names, dates of birth, and email addresses) were not collected within the questionnaire or on the survey platform. For data analysis, Google Forms generated an Excel spreadsheet with the answers to the questionnaires.

#### 2.3.1. Screening Questionnaire

The screening questionnaire collected the following: (1) sociodemographic data, including sex, age, marital status, and current employment status; (2) sporting data, including the type of ultraendurance sport(s) practiced, age at the first ultraendurance event, the number of weekly hours of training, training preferences (with a group and/or alone), the number of ultraendurance events per year, sports club participation (“yes”/“no”), and the consumption of food supplements (“yes”/“no”); and (3) healthcare data, including the type of physician consulted for the medical certificate of fitness and whether the physician had previously discussed ED with the participant (“yes”/“no”).

#### 2.3.2. Exercise Dependence Scale-Revised (EDS-R)

ED was investigated via the French version of the Exercise Dependence Scale-Revised (EDS-R) [57,58]. The EDS-R is a 21-item self-administered questionnaire with 7 subscales based on the DSM diagnostic criteria (tolerance; withdrawal effects; intention effects; lack of control; time; reductions in other activities; and continuance) [59]. For each of the 21 items (“statements”), the rating is given on a 6-point Likert scale, ranging from 1 (never) to 6 (always) according to what best corresponds to the participant. The total score of exercise dependence symptoms is calculated out of a possible 126 (higher scores indicate greater dependence). Additionally, the scale allows participants to be categorized into either at risk for ED (ED+, i.e., 5–6 on the Likert scale for ≥3 subscales), nondependent symptomatic (ED−/S+, i.e., 3–4 on the Likert scale for ≥3 subscales and failing to meet the criteria of ED+), or nondependent asymptomatic (ED−/S−, failing to meet the criteria of ED+ and/or ED−/S+) groups according to the scores on 3 or more of the subscales [59].

#### 2.3.3. Big Five Inventory (BFI)

To assess personality traits, participants completed the French version [60] of the Big Five Inventory (BFI) [61]. The BFI is a valid and reproducible self-administered questionnaire for classifying individuals according to five major personality traits [62]. The BFI is a 44-item questionnaire, and each item is a statement (“I am someone who…” followed by the item statement). The rating is given on a 5-level Likert scale from 1 (strongly disapprove) to 5 (strongly approve). Items are combined into five scales, Extraversion (8 items), Agreeableness (9 items), Conscientiousness (9 items), Neuroticism (8 items), and Openness (10 items), with mean scores based on a 5-point scale for each trait (higher scores indicate higher personality traits).

#### 2.3.4. Screening for Eating Disorders

The screening for possible eating disorders was explored using two items of the SCOFF questionnaire [63] about body image, namely, Fat (“Do you believe yourself to be Fat when others say you are too thin?”) and preoccupation with food, namely, Food (“Would you say Food dominates your life?”). If the participant answers “yes” to both items, possible eating disorders are considered [64].

### 2.4. Statistics

The results are presented as means (standard deviations (SDs)). The normality assumption for quantitative data was verified using a Kolmogorov–Smirnov test. Differences among independent groups (ED+, ED−/S+, and ED−/S−) were tested using a one-way analysis of variance (ANOVA) with Tukey post hoc analysis for quantitative variables and a chi-square test for categorical variables, with a post hoc analysis of Pearson residuals. Regression analysis with an interaction effect was used to check for the confounding impact of possible eating disorders on the significant personality traits associated with ED. Pearson’s correlations were used to determine the strength of the associations between the scores for personality traits and ED and interpreted as 0.11 ≤ r ≤ 0.30 = small, 0.31 ≤ r ≤ 0.49 = moderate, or r ≥ 0.50 = large [65]. The sample size calculation was based on the results of Di Lodovico et al., who found an ED prevalence of 14.2% in endurance athletes [33]; thus, for a target of *n* > 30 in the ED+ group, a minimum sample size of 212 participants was needed. Statistical analyses were performed using SPSS software ver. 25.0 (IBM, Armonk, NY, USA) for Windows, and the significance level was set at *p* < 0.050.

## 3. Results

### 3.1. Characteristics of Participants at Risk for ED

A total of 507 participants (41.6 (9.8) years, 374 men; 73.7%) who participated in a mean of 3.5 (2.1) ultraendurance events each year were included. The mean score was 59.1 (18.7) out of 126 on the EDS-R, and *n* = 37 (7.3%) were classified as at risk for ED (ED+ group.)

The characteristics of participants, with differences between the ED+ and ED− groups, are presented in Table 1. Between the groups, there was no significant difference regarding the demographic characteristics (*p* > 0.050). Participants in the ED+ group trained for a greater duration each week (*p* < 0.001), with 35.2% practicing for at least 15 h per week, whereas this proportion was only 7.5% in the ED− group. There was no difference regarding the type of sport, the age at the first ultraendurance event, the training preference (alone and/or with a group), the number of ultraendurance events per year, the consumption of food supplements, or registration in any sports federation (*p* > 0.050).

Regarding the healthcare characteristics, there was no significant difference regarding the type of physician consulted for the medical certificate of fitness between groups, and general practitioners (GPs) accounted for more than half of the consulted physicians. Interestingly, ED had been more often discussed by GPs in the D+ group than in the D− group (*p* = 0.002) (Table 1).

### 3.2. Characterizing Nondependent Symptomatic Participants (ED−/S+)

**Figure 1:**
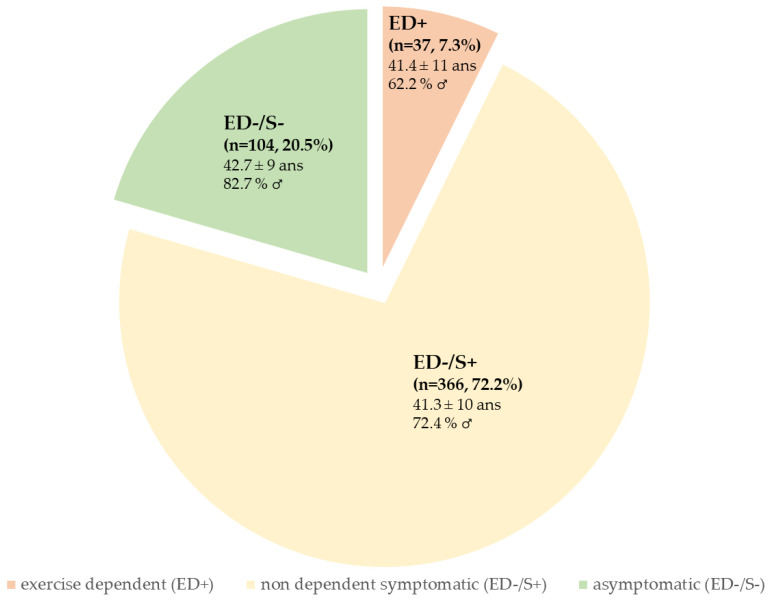
Demographic characteristics of participants in the asymptomatic (ED−/S−), symptomatic (ED−/S+), and exercise-dependent (ED+) groups (*n* = 507).

**Figure 2:**
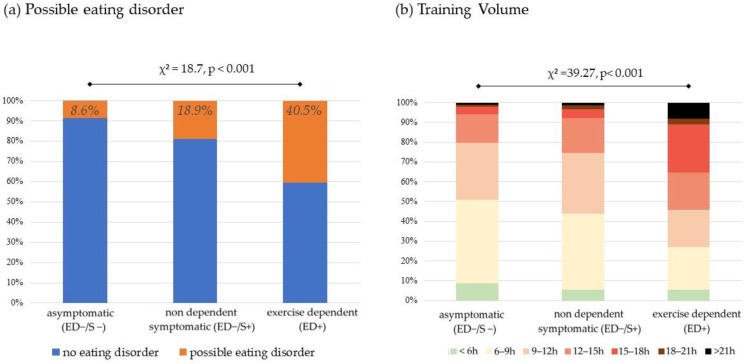
Prevalence of possible eating disorders and training volume in the asymptomatic (ED−/S−), symptomatic (ED−/S+), and exercise-dependent (ED+) groups (*n* = 507).

Among the 470 participants who were not considered at risk of ED, 366 individuals were characterized as nondependent but symptomatic (ED−/S+, 72.2% of the total group), and 104 individuals were characterized as asymptomatic (ED−/S−, 20.5% of the total group). Interestingly, after considering the symptomatic group, there was a significant sex difference (χ^2^ = 7.2, *p* = 0.027), with a greater rate of women in the ED+ group than in the ED−/S− group (37.8% vs. 17.3%) (Figure 1). Additionally, there was a significant difference regarding possible eating disorders, with increased rates among the ED−/S−, ED−/S+, and ED+ groups (8.7%, 18.9%, and 40.5%, respectively, χ^2^ = 18.7, *p* < 0.001) (Figure 2a). Finally, there was a significant difference in terms of training volume with increased training volume per week among the ED−/S−, ED−/S+, and ED+ groups (χ^2^ = 39.27, *p* < 0.001) (Figure 2b).

### 3.3. Association between Personality Traits and ED

The correlations between the total score on the EDS-R scale and the personality traits using the BFI are presented in Table 2. The total score of the EDS-R was positively associated with an almost moderate correlation with neuroticism (r = 0.294; *p* < 0.001). Conversely, we found a significant negative association between the EDS-R score and agreeableness, but with a low correlation coefficient (r = −0.135; *p* = 0.002).

Interestingly, we explored the criteria of ED that were associated with neuroticism and found the highest positive association between neuroticism and the symptoms of “withdrawal” with a moderate correlation (r = 0.398; *p* < 0.001), as shown in Table 3.

**Figure 3:**
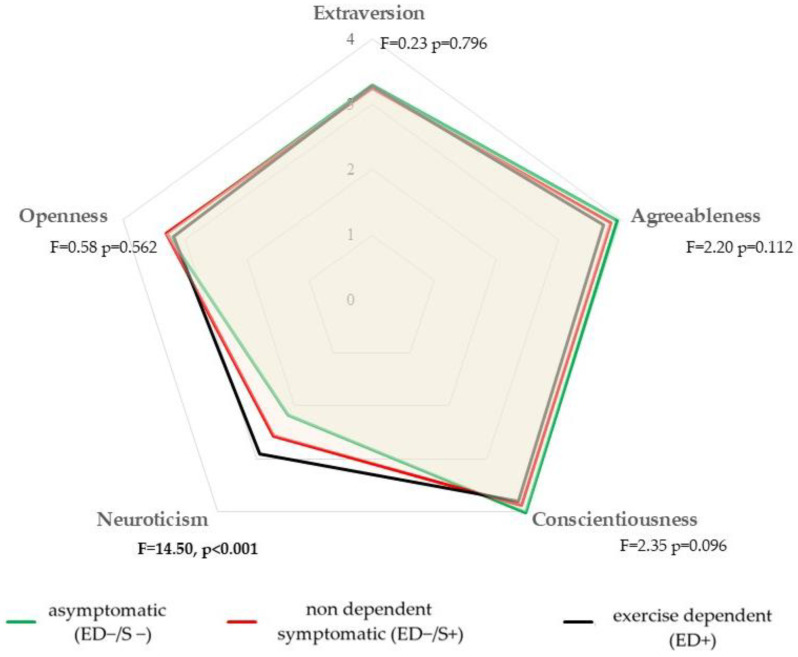
Radar chart comparing the Big Five personality traits among the asymptomatic (ED−/S−), symptomatic (ED−/S+), and exercise-dependent (ED+) groups (*n* = 507).

The graphical representation of the personality traits on the BFI among the ED+, ED−/S+, and ED−/S− groups is presented in Figure 3. The neuroticism score was significantly different among the groups, with greater scores for the ED+ and ED−/S+ groups than for the ED−/S− group (F = 14.50, *p* < 0.001).

**Figure 4:**
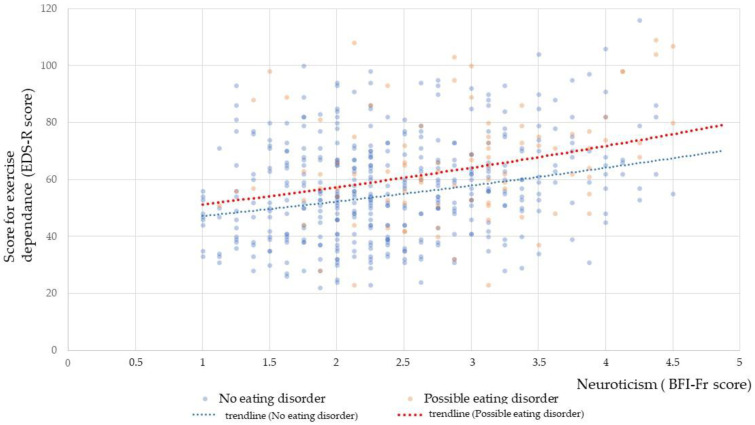
Graphical representation of the association between EDS-R scores and neuroticism scores among participants with or without possible eating disorders (*n* = 507).

Participants with possible eating disorders had higher levels of neuroticism than participants without possible eating disorders (2.92 (0.83) vs. 2.42 (0.78), t = 5.482, *p* < 0.001). Furthermore, we investigated the interaction effect of neuroticism*eating disorder to explain ED by testing the linear regression model: ED (EDS-R score) = x + β*neuroticism (BFI score) + β*eating disorder (no = 0, yes = 1) + neuroticism*eating disorder. This model was significant (F = 19.36, *p* < 0.001), but only neuroticism was largely associated with ED (F = 28.52, *p* < 0.001), in contrast to eating disorder (F = 0.11, *p* = 0.918) and neuroticism*eating disorder (F = 0.62, *p* = 0.431). If a coexisting eating disorder was associated with systematically increased EDS-R scores, it did not influence the association between neuroticism and ED, with neuroticism being the strongest predictor associated with increased EDS-R scores (Figure 4).

## 4. Discussion

This is the first study to evaluate the personality traits associated with exercise dependence among ultraendurance athletes, and the main finding was that ultraendurance athletes with ED have higher neuroticism than those without ED, regardless of the presence of possible eating disorders.

These results are in accordance with the findings from previous studies not specific to endurance sports, which found that high neuroticism [66,67,68,69] was associated with the risk of ED. Neuroticism is defined as a persistent tendency to experience negative emotions while being aware of psychological suffering (neurosis) and exhibiting recurrent nervousness. Reactions to threats, frustrations, and losses can range from a high level of intensity in the face of a minor obstacle to a minimal reaction to a major difficulty. This personality trait represents a major challenge in terms of public health. Indeed, it is strongly correlated with numerous mental disorders and various comorbidities and generates a high rate of recourse to general and psychiatric medicine [70]. The processes underlying the trait of neuroticism are still debated, with some authors suggesting an essentially acquired environmental [71], innate and genetic [72], or more likely mixed origin [73]. Furthermore, among the genetic features associated with neuroticism, certain genomic regions concern dopaminergic neuroblasts and brain reward circuits [74], which are also involved in the development of ED [68]. Studies have found a link between neuroticism and a tendency to experience more stress [75]. Some authors have introduced the possibility of “healthy neuroticism” with a high level of neuroticism combined with a high level of conscientiousness corresponding to circumstances under which neuroticism is associated with positive outcomes, including regular physical activity [76]. In contrast, the present results showed that low conscientiousness was not significantly associated with high neuroticism in participants with ED. To a lower extent, greater ED scores were associated with lower scores for agreeableness, which is characterized as displaying less prosocial behavior and more rejection cues and hostile emotions [77]. Interestingly, the high neuroticism/low agreeableness phenotype has a stronger predictive value for genetic vulnerability to depression than neuroticism alone [78]. Additionally, it has been stipulated that neuroticism and agreeableness could interact to predict the intensity of symptoms and that the neuroticism–symptom relationship was pronounced at low levels of agreeableness [79].

Previous studies have explored the personality traits associated with substance use disorders and behavioral addictions using the five-factor model. Interestingly, they found similar patterns of personality traits with high neuroticism and low agreeableness in individuals with alcohol, tobacco, and marijuana use disorders but also in individuals with social media and gambling dependence [49]. These findings corroborate the evidence that individuals with ED share characteristics similar to those found in more conventional addictions, which would help to support the characterization of ED as a behavioral addiction [10]. This could draw the attention of sports specialists and/or healthcare professionals to give specific attention to the history of addiction in individuals who plan to engage in ultraendurance sports and to identify possible coexisting addictions in individuals who are at risk of ED. Indeed, several studies have demonstrated that ultraendurance athletes experience mental health issues [80,81], but the causal effect remains uncertain, especially if participants use sports as a primary coping skill or excessive compensatory behavior [82].

Personality traits demonstrate both continuity and change over time and can serve both as relatively stable predictors of consequential outcomes of success and as actionable targets for intervention [45,83]. Thus, personality traits could be key to the development and maintenance of addictive behavior [84], but some evidence suggests that personality could predispose an individual to these behaviors and also be the result of addictive disorders, and this relationship remains to be determined more precisely [85]. Several models are based on the principle that individual differences in personality traits or a person’s core characteristics reflect variations in sensitivity to positive or negative reinforcement and could be key to the development of addictions [86,87]. However, identifying the mediating role of neuroticism in the development of ED remains challenging. It has been suggested that neuroticism and negative coping strategies could mediate the relationship between negative affect and behavioral addictions [88,89,90,91]; thus, engaging in exercise could be a coping strategy to alleviate negative emotions [67,92,93]. Exercising has been described as a healthy and virtuous coping strategy for individuals with mental disorders such as depression or anxiety, mostly due to the mental health benefits of physical activity [5]. However, neuroticism, as with other personal and situational parameters, could be an important factor underlying the difference between using exercise as a healthy or maladaptive coping strategy [92,94]. Similarly, Golshani et al. found that mental toughness could moderate the association between heavy exercising and greater mental health issues in that lower mental toughness was associated with worse mental health issues among heavy exercisers [32]. Interestingly, mental toughness was negatively associated with neuroticism in ultraendurance athletes [56], Moreover, neuroticism could heighten negative affect and explain the maintenance of unhealthy behavior, which is supported by the association and moderate positive correlation between neuroticism and the withdrawal effect [90,95,96].

Our results support the characterization of a continuum in the development of ED [42,97]. Indeed, the findings highlighted progressively increased levels of neuroticism, greater rates of possible coexisting eating disorders, and increased training volumes among asymptomatic, symptomatic, and exercise-dependent individuals. Freimuth et al. proposed four phases to distinguish exercise addiction from other forms of intense and frequent exercise behaviors [98]. According to this continuum, recreational exercise can progress to at-risk exercise behavior when athletes increase the frequency and intensity, especially for the mood-altering effect of exercise, including improved self-esteem, the relief of stress, or coping [97]. Then, problematic exercise is indicated when secondary negative adverse events are predominant with uncontrollable consequences, and finally, exercise addiction is characterized as worsening direct and secondary consequences with impairments in daily functioning and the inability to meet social responsibility [42]. Thus, the early identification of participants with at-risk exercise behavior, especially with the use of personality traits, could help to avoid progression to ED.

Our results indicated greater rates of possible eating disorders among participants at risk for ED (40.5%) than in those who were asymptomatic for ED (8.7%). There is substantial evidence that eating disorders are frequent coexisting disorders associated with ED and are considered either a causal factor (“secondary addiction”) or a consequence of ED [25]. Interestingly, although neuroticism was higher in individuals with eating disorders, the interaction effect did not reveal that the association between neuroticism scores and ED depends on the presence of possible eating disorders. These results are in contrast with those of Bamber et al., who found that women at risk of primary ED did not exhibit the same personality characteristics and levels of psychological distress as those with coexisting eating disorders [69]. Our results suggested that neuroticism could be a common personality trait associated with both eating disorders and ED and that it should be considered independently, e.g., regardless of the presence of coexisting eating disorders, for the management of ED. However, we did not assess

The participants at risk for ED were more likely to have discussed this topic with their GPs. As GPs are more prone to see athletes in primary care and for fitness certification, it is important to screen athletes for ED risk. Additionally, GPs are more aware of personal and family risk factors for behavioral addiction, including ED. Nevertheless, regardless of whether GPs are aware of ED risk, more than 60% of athletes who met the ED criteria had never discussed this with their GPs, and it is unknown what makes GPs consider the possibility of ED. It is very likely that a very high training volume or its related injuries might be a key for the consideration of ED but are not sufficient; better knowledge of the continuum of ED, better identification of the risk factors (here, personality traits), and the use of appropriate screening tools are mandatory [43].

As expected, we found an increased training volume in the asymptomatic, symptomatic, and exercise-dependent groups. If ED is not restricted to frequent exercise, it confirms that these athletes may be exposed to acute and long-term health issues associated with an extreme volume of exercise [41,99]. As ED characteristics are continuous despite health issues or injuries, it is crucial to help athletes monitor their training volume during the treatment of ED or after high-volume-induced injuries.

Finally, these results could help in designing therapeutic interventions for individuals with ED. Cognitive–behavioral therapy is one of the most common approaches in the treatment of ED alone or as part of combination treatment strategies [97]. This approach aims to identify and modify dysfunctional cognitions to modify negative emotions and behaviors; thus, individuals at risk for ED could then be provided with interventions tailored to reducing neuroticism [70,100]. Additionally, it has been described that obsession with and dedication to sports could bear a strong relationship with ED and that harmonious passion is positively related to positive affect, while it is negatively linked to negative affect [101]. Thus, varying sports participation without decreasing the time spent on other important life activities could help increase the pleasure from and enjoyment of exercise.

### 4.1. Limitations

The current study has a number of limitations. First, this was a web-based questionnaire that was distributed through federal associations, ultraendurance event organizers, and social networks. Thus, we could not control the total population, the rate of completion, or potential selection bias. However, the rate of athletes at risk for ED was consistent with that reported in the literature [33]. Second, we used only two of the five items of the SCOFF questionnaire to explore the presence of possible eating disorders; however, the items chosen had a strong item-total correlation [102]. Finally, we previously stated that personality demonstrates both continuity and change over time and can be influenced by fluctuating physical and psychological states. In this context, it would have been interesting to assess other factors that could influence personality, such as depression, anxiety, sleep disturbances, low self-esteem, and also socioeconomic status.

### 4.2. Future Research Direction

The present study highlighted that the personality trait of neuroticism was associated with the risk of ED in ultraendurance sports. Further research should test the effectiveness of specific behavioral interventions or mindfulness-based cognitive therapy to tackle neuroticism [100,103] for the prevention and treatment of ED in ultraendurance athletes, as mentioned above. Furthermore, it would be interesting to test the association of neuroticism not only with the risk of ED but also with the co-occurrence of adverse physical and psychological effects of ED [30,31]. Additionally, compulsivity and impulsivity represent important dimensions of non-substance addictions [104], and a previous study found coexisting higher neuroticism and increased impulsivity in ED [68]. Further studies are needed to explore the relationship between neuroticism and impulsivity in this population. Finally, we have explored ED in endurance athletes, but the risk of ED has also been reported in resistance-trained athletes [105], which are also exposed to body dysmorphic disorder and muscle dysmorphia [106,107]. Future studies should compare the personality traits and mechanisms underlying ED between endurance- and resistance-trained athletes.

## 5. Conclusions

Ultraendurance athletes at risk of ED have a higher level of neuroticism, as in numerous other SUDs or behavioral addictions, which was associated with the symptom of withdrawal. Neuroticism is related to negative emotions, and it can be hypothesized that excessive exercise could be a maladaptive coping strategy to alleviate negative affect and lead to ED. Possible eating disorders were more frequent in participants at risk of ED but did not influence the association between neuroticism and ED. The early identification of ultraendurance athletes at risk of ED with personality trait recognition or coexisting risk factors could help to better prevent the occurrence and worsening of symptoms and better manage their health.

## Figures and Tables

**Table 1:** (**a**) Characteristics of participants and differences between individuals at risk of ED and those not at risk (*n* = 507). (**b**) Characteristics of participants and differences between individuals at risk of ED and those not at risk (*n* = 507).

(**a**)					
		No exercise dependence	Exercise dependence	Differences between groups	
		*n* = 470	*n* = 37	χ^2^ or *t*-test	*p*-value
Age (years, (SD))		41.6 (9.7)	41.4 (11.1)	0.155	0.877
Age at first ultra (years, (SD))		33.4 (8.3)	34.7 (8.4)	−0.89	0.375
Sex (*n* (%))	Male	351 (74.7%)	23 (62.2%)	2.78	0.096
	Female	119 (25.3%)	14 (37.8%)		
Marital status (*n* (%))	Single	107 (22.8%)	13 (35.1%)	2.91	0.088
	Married	363 (77.2%)	24 (64.9%)		
Employment	Managers	192 (40.9%)	15 (40.5%)	3.663	0.886
	Intermediate job	103 (21.9%)	5 (13.5%)		
	Services/sales workers	71 (15.1%)	8 (21.6%)		
	Business owners	47 (10.0%)	4 (10.8%)		
	Laborer	25 (5.3%)	2 (5.4%)		
	Retired	13 (2.8%)	2 (5.4%)		
	Students	10 (2.1%)	1 (2.7%)		
	Unemployed	7 (1.5%)	0 (0%)		
	Agricultural workers	2 (0.4%)	0 (0%)		
Type of sport (*n* (%))	Running—Trail running	199 (42.3%)	11 (29.7%)	6.236	0.284
	Multiple	104 (22.1%)	7 (18.9%)		
	Triathlon	104 (22.1%)	10 (27.0%)		
	Cycling	57 (12.1%)	9 (24.3%)		
	Swimming	5 (1.1%)	0 (0.0%)		
	Skiing	1 (0.2%)	0 (0.0%)		
Training volume (h per week (SD))	Below 9 h	214 (45.5%)	10 (24.0%)	36.51	<0.001
	9–15 h	221 (47.0%)	14 (37.8%)		
	Above 15 h	35 (7.5%)	13 (35.2%)		
Sport club participation (*n* (%))	No	225 (47.9%)	17 (45.9%)	0.051	0.821
	Yes	245 (52.1%)	20 (54.1%)		
Training preferences (*n* (%))	Alone	113 (24.0%)	7 (18.9%)	0.95	0.622
	Group	5 (1.1%)	0 (0.0%)		
	Both	352 (74.9%)	30 (81.1%)		
Participation in ultraendurance events per year (*n* (SD))		3.1 (2.5)	2.9 (1.5)	0.457	0.648
Possible eating disorder (*n* (%))	No	392 (82.4%)	22 (59.5%)	13.13	<0.001
	Yes	78 (16.6%)	15 (40.5%)		
(**b**)					
		No exercise dependence	Exercise dependence	Differences between groups	
		*n* = 470	*n* = 37	χ^2^	*p*-value
Who signed the fitness certificate?					
	General Practitioner (GP)	266 (56.6%)	19 (51.4%)	4.489	0.481
	Sports Physician	147 (31.3%)	13 (35.1%)		
	Cardiologist	33 (7.0%)	2 (5.4%)		
	Myself or Friends	14 (3.0%)	1 (2.7%)		
	Occupational physician	4 (0.9%)	0 (0.0%)		
	None	6 (1.3%)	2 (5.4%)		
Did physicians ever talk about ED?					
	No	397 (84.5%)	24 (64.9%)	9.358	0.002
	Yes	73 (15.5%)	13 (35.1%)		
Do you take food supplements?					
	No	121 (25.7%)	12 (32.4%)	0.793	0.373
	Yes	349 (74.3%)	25 (67.6%)		

**Table 2:** Correlation between personality traits (BFI) and the total score of exercise dependence (EDS-R) (*n* = 507).

	Extraversion		Agreeableness		Conscientiousness		Neuroticism		Openness	
EDS-R score	Pearson’s *r*	*p*-val	Pearson’s *r*	*p*-val	Pearson’s *r*	*p*-val	Pearson’s *r*	*p*-val	Pearson’s *r*	*p*-val
	−0.031	0.487	−0.135	0.002	−0.062	0.165	0.294	<0.001	−0.012	0.779

EDS-R: Exercise Dependence Scale-Revised; *p*-val: *p*-value.

**Table 3:** Correlation between scores on the neuroticism scale (BFI) and the symptom subscales of exercise dependence (EDS-R) (*n* = 507).

	Tolerance		Withdrawal		Continuance		Lack of Control		Reduction in Activities		Time		Intention Effect	
EDS-R score	Pearson’s *r*	*p*-val	Pearson’s *r*	*p*-val	Pearson’s *r*	*p*-val	Pearson’s *r*	*p*-val	Pearson’s *r*	*p*-val	Pearson’s *r*	*p*-val	Pearson’s *r*	*p*-val
	0.089	0.045	0.398	<0.001	0.183	<0.001	0.198	<0.001	0.244	<0.001	0.172	<0.001	0.213	<0.001

EDS-R: Exercise Dependence Scale-Revised; *p*-val: *p*-value.

## Data Availability

The data that support the findings of this study are available upon request from the corresponding author, J.D.

## Supplementary Materials

The following supporting information can be downloaded at: https://www.mdpi.com/article/10.3390/ijerph20021042/s1, Table S1: List of the federations/event/social media groups targeted for the survey.
