# Supplementary material for: Personality Traits Associated with the Risk of Exercise Dependence in Ultraendurance Athletes: A Cross-Sectional Study
Source: Int J Environ Res Public Health. 2023 Jan 6;20(2):1042. doi: 10.3390/ijerph20021042 (PMC9858902; doi:10.3390/ijerph20021042)
Supplement: Supplementary file 1 [file ijerph-20-01042-s001.zip › ijerph-2104539-supplementary.pdf]

International Journal of
Environmental Research
and Public Health

MDPI

Table S1: List of the federations/event/social media groups targeted for the survey.

**National Sports Federations**	Fédération Française d'Athlétisme (athletics)
	Fédération Française de Triathlon (triathlon)
	Fédération Française de Natation (swimming)
	Fédération Française de Cyclisme (cycling)
**Ultraendurence events**	Ultra Trail Mont Blanc (UTMB) (mountain ultramarathon)
	Ultra Trail scientifique Clécy (UTCS) (mountain ultramarathon)
	Triathlon International de Deauville (triathlon)
	La Barjo Ultra Trail (mountain ultramarathon)
	Ch'TriMan Gravelines (triathlon)
	FrenchMan Triathlon (triathlon)
	Triathlon de la Madeleine (triathlon)
	T24 Xtrem Triathlon (triathlon)
	BearMan Xtri (triathlon)
	Triathlon de l'Alpe d'Huez (triathlon)
	Troll Enez Swimrun (swimrun)
	Gravelman Series (cycling)
	Ironlakes (triathlon)
	La Baroudeuse Road RACE / TransAlpes (cycling)
	Bordeaux-Paris Ultra Cycling Challenge (cycling)
	Asics Sainté-Lyon (ultramarathon)
	TransContinental Race (cycling)
	L'Ultra Marin (ultramarathon)
	Gravel Tro Breizh (cycling)
	IronMan France (triathlon)
	EmbrunMan Triathlon (triathlon)
	La Diagonale des Fous (mountain ultramarathon)
	BayMan Triathlon (triathlon)
	Normandicat (cycling)
	AlpsMan Triathlon (triathlon)
	VercorsMan Triathlon (triathlon)
	L'Endu'Rance Trail des Corsaires (ultramarathon)
	L'Ultratour des 4 massifs (Ut4M) (mountain ultramarathon)
**Social networks groups**	La Clinique du Coureur (running)
	Ride Across France (cycling)
	Trail je suis addict (mountain ultramarathon)
	Bref, je fais du triathlon (triatlon)
	Ultracycling Aventure (cycling)
	Baroudeur Team groupe privé (cycling)
